# Atypical Parathyroid Tumor: A Case Report on the Diagnostic and Therapeutic Approach

**DOI:** 10.7759/cureus.104215

**Published:** 2026-02-25

**Authors:** Sofia Theofilopoulou, Theodoros Sidiropoulos, Panagiotis Kokoropoulos, Spyridon Christodoulou, Efthimis Poulios

**Affiliations:** 1 Medical School, Attikon University Hospital, University of Athens, Athens, GRC; 2 4th Department of Surgery, Attikon University Hospital, University of Athens, Athens, GRC

**Keywords:** atypical parathyroid adenoma, parathyroid adenoma, parathyroid carcinoma, parathyroidectomy, primary hyperparathyroidism

## Abstract

Atypical parathyroid tumors represent an intermediate entity between parathyroid adenoma and carcinoma and constitute a rare cause of primary hyperparathyroidism. They are characterized by equivocal histological findings, while lacking evidence of malignant involvement. Diagnosis remains challenging and requires both clinical and histopathological correlation. We present the case of a 60-year-old man with a history of gastrointestinal stromal tumor (GIST) treated in 2022 with partial gastrectomy and partial hepatectomy. During routine follow-up, elevated calcium and parathyroid hormone levels were detected. Ultrasound revealed a left inferior parathyroid adenoma measuring 16 mm in diameter. The patient underwent parathyroidectomy. Histological examination demonstrated a tumor with atypical features, consistent with an atypical parathyroid adenoma. There is no evidence of recurrence two and a half years after the adenoma was removed, and the patient remains under surveillance.

Atypical parathyroid tumors exhibit features such as nuclear atypia, increased mitotic activity, and fibrous capsule thickness, without the clear vascular or local invasive behavior characteristic of carcinoma. The differential diagnosis between adenoma and carcinoma is difficult but crucial for prognosis and postoperative management. Surgical excision is the treatment of choice, while close postoperative surveillance is necessary due to the risk of recurrence. This case highlights the importance of systematic evaluation of hypercalcemia and the crucial role of histological analysis in diagnosing atypical parathyroid tumors. Despite their relatively favorable prognosis, long-term follow-up is essential for early detection of recurrence.

## Introduction

Primary hyperparathyroidism (PHPT) is the third most common endocrine disorder, following thyroid cancer and diabetes. In earlier years, patients with hyperparathyroidism primarily presented with osteitis fibrosa cystica and kidney stones; thus, the disease was first described as 'osteitis with renal calculi.' It was subsequently linked to the parathyroid glands around 1930 [1]. Later, the disease was also found to cause polyuria and polydipsia as well as peptic ulcers, neuromuscular disorders, muscle atrophy, vomiting, nausea and constipation, hypertension, and, in some cases, even acute pancreatitis was observed [2-4]. Manifestations in the central nervous system vary from psychiatric conditions to consciousness disorders, even coma [3].

While primary hyperparathyroidism is commonly diagnosed incidentally via routine laboratory screening in developed countries, patients in developing countries often present with symptomatic skeletal complications [5,2].

PHPT can occur sporadically or as part of genetic syndromes such as multiple endocrine neoplasia type I, type IIA, type IV (MEN1, MEN2A, and MEN4), and hyperparathyroidism-jaw tumor syndrome (HPT-JT). Usually, syndromic PHPT is associated with multiple parathyroid tumors and can be either familial or non-hereditary [5]. At a molecular level, more than 50% of familial cases of parathyroid carcinogenesis, and over 75% of sporadic ones, are associated with mutations in the *CDC73* tumor suppressor gene [6].

Tumors causing primary hyperparathyroidism can range from benign hyperplastic processes to carcinomas. Around 80-85% of tumors are adenomas, followed by primary parathyroid hyperplasia (around 15%), while less than 1% are parathyroid carcinomas [7,8]. Benign adenomas are more often located in the lower glands than in the upper, and they usually present as smooth red or brown nodules surrounded by a capsule [7]. Parathyroid carcinomas are usually suspected when serum calcium levels exceed 14-15 mg/dL, while benign adenomas can present with only mild hypercalcemia or even normocalcemia. Imaging cannot differentiate between the two, and fine-needle aspiration biopsy (FNAB) is not recommended as it carries the risk of needle-track seeding. The diagnosis is mostly reached after the parathyroidectomy since surgical removal is considered the established treatment [9].

Atypical parathyroid adenoma is a rare entity associated with primary hyperparathyroidism in a percentage of 1% or less [10,11]. The average weight of those adenomas is around 1g, while a normal parathyroid gland usually weighs 0.5g with overall dimensions around 5×3×1 mm [12]. The current 2022 World Health Organization (WHO) classification suggests the use of “atypical parathyroid tumor” instead of “atypical adenoma” since it is now considered a borderline tumor whose potential for malignancy remains uncertain. Therefore, atypical parathyroid tumors share a lot of characteristics with parathyroid carcinomas [9]. Common features include clinical phenotype, biochemical profile, and histopathology (fibrosis, increased mitotic activity, and capsule thickening). In atypical adenomas, however, both vascular and perineural invasion, lymph node enlargement, and distant metastasis are absent [13]. The question that is yet to be answered is whether atypical parathyroid tumors are an initial stage of parathyroid carcinomas [5].

The purpose of presenting this case with a rare cause of primary hyperparathyroidism is to emphasize the importance of considering this entity in the differential diagnosis both due to its diagnostic challenges and its need for follow-up. 

## Case presentation

A 60-year-old male presented with primary hyperparathyroidism in the 4th Department of Surgery, Attikon University Hospital, Athens, Greece, in September 2023. Blood examination revealed elevated Calcium at 10.4 mg/dl (Normal range: 8.4-10.2 pg/ml) and parathyroid hormone (PTH) at 123.4 pg/ml (Normal values: 18.5 - 88 pg/ml). He underwent a partial gastrectomy and partial hepatectomy for metastatic GIST in 2022. His past medical history is otherwise unremarkable. 

**Figure 1:**
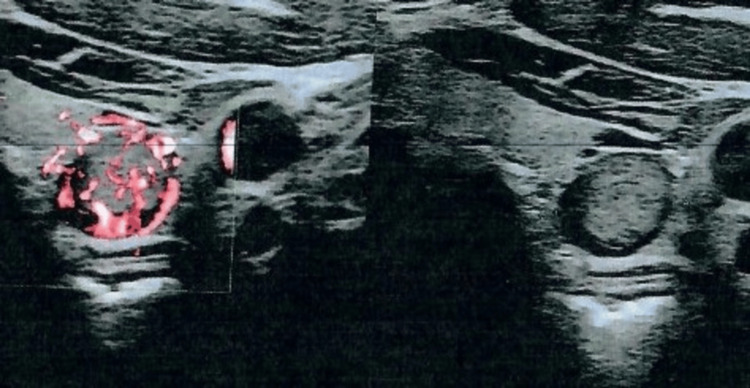
Ultrasound showing a hypoechoic lesion of 16x13.2x8 mm between the mid-portion and the inferior pole of the left thyroid lobe

He did not report any symptoms. Ultrasound showed a hypoechoic lesion of 16x13.2x8 mm between the mid-portion and the inferior pole of the left thyroid lobe, possibly suggesting a left low parathyroid adenoma. Lymph nodes within the examined area appeared normal (Figure 1).

**Figure 2:**
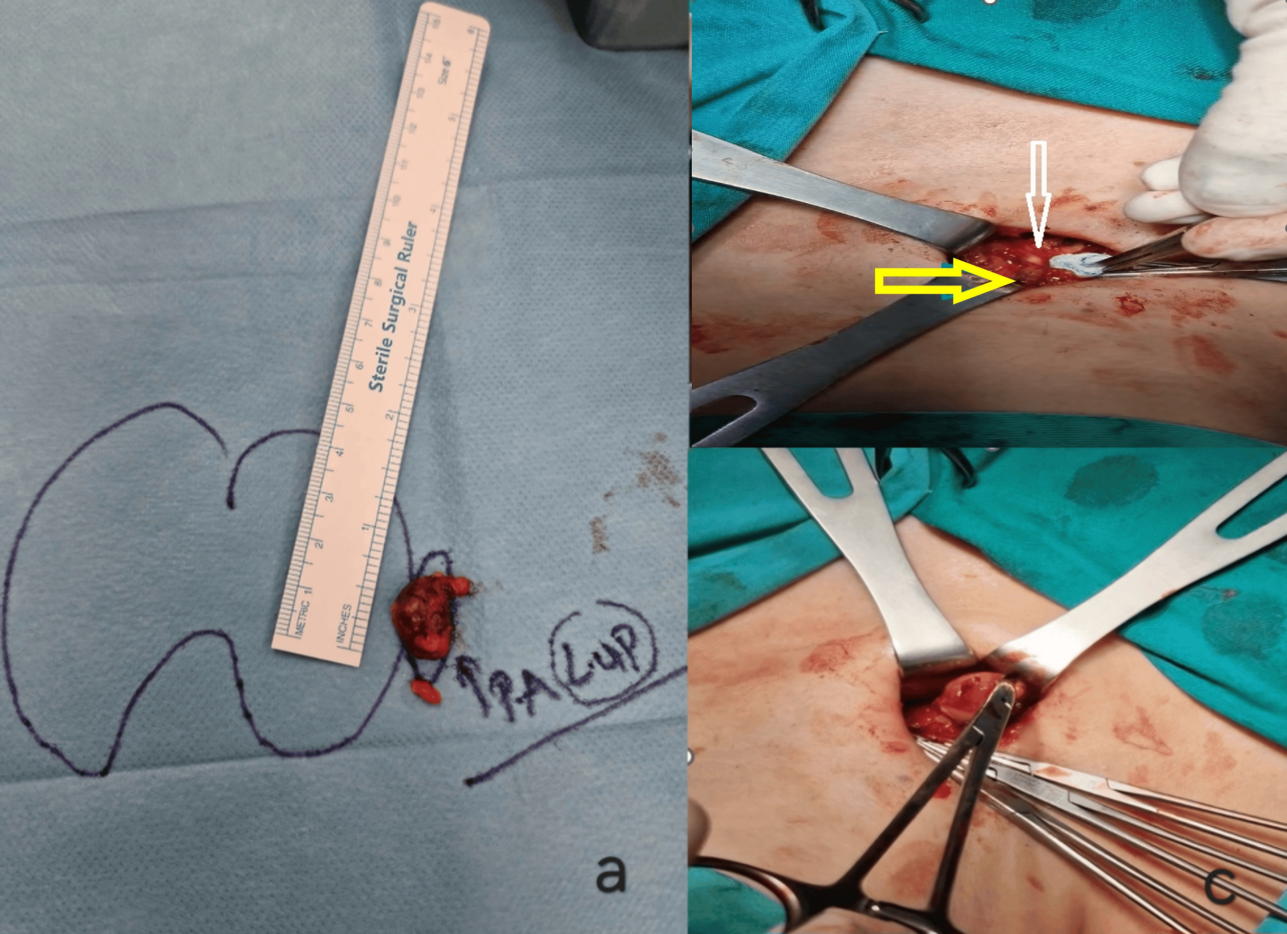
Macroscopic evaluation of atypical parathyroid tumor 2a. Macroscopic appearance of the atypical parathyroid adenoma, illustrating its size. 2b. Yellow arrow: Upper parathyroid gland; White arrow: Right recurrent laryngeal nerve. 2c. Intraoperative photo of a mosquito clamp holding the lesion."

The patient was scheduled for a left low parathyroidectomy. His pre-surgical intact PTH (iPTH) was high (114 pg/mL). Under general anesthesia, a localized layered dissection was performed with preservation of the left recurrent laryngeal nerve. The nodule was located and confirmed as parathyroid tissue via frozen section analysis (Figure 2). Intraoperative PTH dropped to 65 pg/ml 10 minutes post-excision, confirming removal of the adenoma. Patient recovered quickly and was discharged the following day.

**Figure 3:**
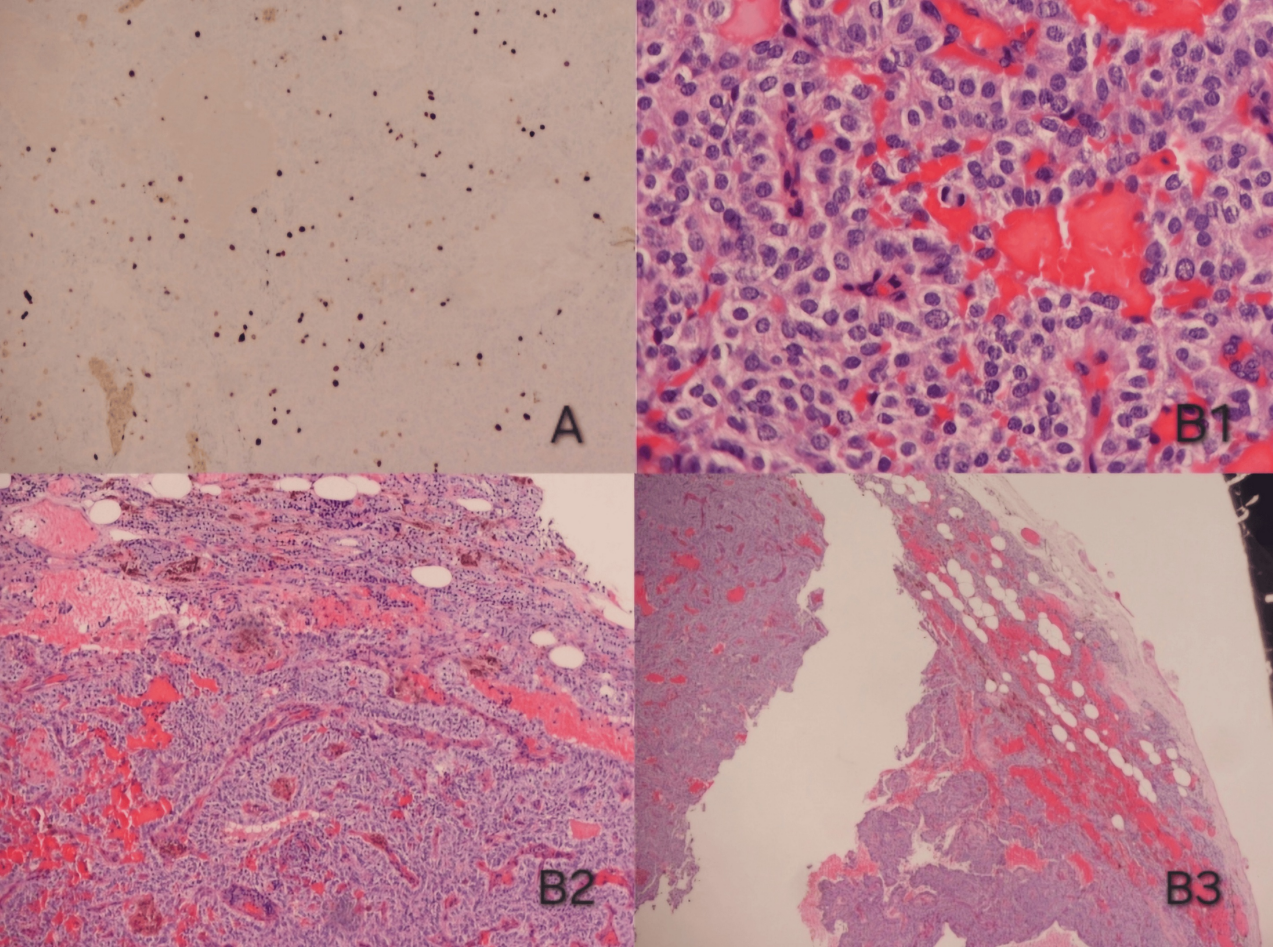
Histopathological and immunohistochemical features of atypical parathyroid tumor A. Ki-67 immunostain showing low proliferative activity. B1,2,3. Hematoxylin – Eosin stain showing mitotic potential (6 mitosis/10 mm2) and characteristics of atypical parathyroid tumor.

Microscopic examination identified parathyroid cells demonstrating cytologic atypia, mild pleomorphism, and an increased mitotic potential (6 per 10 mm^2)^. However, there was also a strip of parathyroid tissue with well-preserved architecture and normal chief cells. In immunochemistry, the Ki67 proliferation index was really low (2%). Cells were found positive for Chromogranin A, GATA binding protein-3 (GATA-3), and Cytokeratin AE1/AE3 (CKAE1/AE3), and negative for Thyroid Transcription Factor-1 (TTF1), Thyroglobulin, S100, and Galectin 3. The diagnosis was an atypical parathyroid lesion of uncertain malignant potential (Figure 3). 

At 2.5-year follow-up, the patient remains recurrence-free, with serial ultrasound imaging and biannual calcium and PTH levels maintaining within normal ranges.

## Discussion

The preferred therapeutic approach for symptomatic primary hyperparathyroidism is a parathyroidectomy, with the surgical scope adjusted based on the underlying etiology [2]. In benign adenomas, imaging is used to pinpoint the exact location of the pathological parathyroid gland. Usually, single-photon emission computed tomography (SPECT/CT) and neck ultrasound with Color Doppler are used [9]. Like in our case, if there is a clear image of the diseased gland, then a minimally invasive parathyroidectomy is performed. On the contrary, if there is a suspicion of multiglandular disease or clear visualization is not achieved, then a bilateral neck exploration is preferred [8]. Key operative landmarks include the junction of the recurrent laryngeal nerve and the inferior thyroid artery at the cricothyroid articulation, situated 1 cm below the superior parathyroid gland. In most patients, the two sides of the parathyroid gland have mirror symmetry [9]. From imaging studies of our patient, the disease was only located in the left lower parathyroid, and we proceeded with minimal invasive parathyroidectomy.

In cases of parathyroid carcinomas, the surgical management is more extensive [6]. Ideally, an en bloc resection is performed [14]. It is considered R0 when there is no capsular disruption. If the tumor invades the recurrent laryngeal nerve, it should be resected as well, while central regional lymph node clearance should only be performed when nodal involvement is suspected. Usually, this accounts for 6.5-32% of patients. In cases of adhesions, ipsilateral thyroid lobo-isthmectomy should be combined with en bloc resection [9]. Chemotherapy and radiotherapy are only used in lack of other alternatives for non-surgical patients [11]. Neither is indicated routinely, unless excision is incomplete, requiring a discussion on adjuvant radiotherapy. Both physicians and patients should take into account the risk of radiotherapy-related fibrosis [14].

The management of atypical parathyroid tumors is not well established due to the rarity of the lesion. Some studies recommend following the treatment principles applied to parathyroid carcinomas [15]. Contrasting with other presentations, the patient's age and ultrasound characteristics suggested a benign adenoma, leading to our decision for a focused excision of the solitary diseased gland.

In cases of parathyroid carcinomas or atypical parathyroid adenomas in the spectrum of MEN syndrome, the surgical procedure is different. In most patients, a subtotal parathyroidectomy removing at least 3.5 glands is the first choice of treatment [16]. Total parathyroidectomy with auto-plantation should also be considered for extensive lesions. Normally, when the excision is R0, both in parathyroid carcinomas and atypical adenomas, PTH levels should return to normal right after excision [9]. If PTH levels stay elevated or hypercalcemia is found, then post-treatment imaging should be performed, including cervical ultrasound, injected cervico-thoracic CT, and/or a cervical MRI [9,14].

Usually, for parathyroid carcinomas, the recurrence rate is between 30 and 67% [14]. Recurrence rates for atypical parathyroid adenomas differ across studies. Some studies range report recurrence rates 0 to 3.7% while others suggest a 10% rate [10,14,15,17]. Risk factors include age < 47 or > 65 years, calcemia >150 mg/L, PTH concentration of > 700 pg/ml, tumor size > 3 cm, N1 status, absence of en bloc resection, vascular invasion, and R1 excision. Metastasis of parathyroid cancer usually involves the lungs, bones, and liver, and it is treated either with anthracycline-based chemotherapy or with targeted anti-angiogenic therapy [14]. Metastasis in cases of atypical adenomas has not been described yet. Patients with atypical adenomas should have specialized and long-term follow-up, with at least annual measurement of blood calcium and PTH [10]. In our case, we recommend the measurement of PTH and calcium every six months, and an ultrasound every twelve months.

Given the rarity of the disease, physicians receive treatment data only from case reports and small cohort studies [17]. Out of the 169 case reports that show up on the PubMed search of “atypical parathyroid adenomas”, only 44 refer to the lesion. The patient we present gives an insight into a case of atypical adenoma treated as an adenoma and not as a carcinoma with an uneventful recovery, and no recurrence during ongoing follow-up. Although atypical adenoma presents several differences compared to common adenoma, as observed in the histology report of our patient, it does not seem to have the potential to metastasize, and a complete parathyroidectomy is adequate. The surgical management was performed in line with the established guidelines referring to parathyroid adenomas, and close follow-up showed no recurrence or metastasis. Follow-up is necessary because there is a possibility of recurrence. Following the surveillance approach used in major studies [5,10], we will monitor patients for 5 years after resection.

## Conclusions

Atypical parathyroid adenomas represent a rare entity that lies between benign adenomas and parathyroid carcinomas, making accurate diagnosis difficult preoperatively, as demonstrated in our case. Complete surgical excision remains the primary treatment, while long-term follow-up is essential given the rates of recurrence. Nevertheless, there is a great need for larger studies to establish comprehensive clinical guidelines and to provide physicians with a framework, so that they understand better the disease and improve patient outcomes.
